# Supplementary material for: A modular platform for automated cryo-FIB workflows
Source: eLife. 2021 Dec 24;10:e70506. doi: 10.7554/eLife.70506 (PMC8769651; doi:10.7554/eLife.70506)
Supplement: Supplementary file 1. [file elife-70506-supp1.docx]

| **Sample** | **Micro-expansion joint milling** | | | | **Rough milling** | | | | **Fine milling** | | | |
| --- | --- | --- | --- | --- | --- | --- | --- | --- | --- | --- | --- | --- |
|  | distance from lamella [μm] | width [μm] | current [nA] | time [s] | step | nominal lamella thickness [μm] | current [nA] | time [s] | step | nominal lamella thickness [nm] | current [pA] | time [s] |
| Sum159 | 4 | 0.3 | 1 | 30 | 1 | 5 | 1 | 480 | 1 | 400 | 100 | 210 |
|  |  |  |  |  | 2 | 3 | 0.5 | 210 | 2 | 300 | 50 | 150 |
|  |  |  |  |  | 3 | 1 | 0.3 | 210 |  |  |  |  |
| HeLa | 4 | 0.3 | 1 | 30 | 1 | 5 | 1 | 540 | 1 | 300 | 50 | 360 |
|  |  |  |  |  | 2 | 3 | 0.5 | 300 |  |  |  |  |
|  |  |  |  |  | 3 | 1 | 0.3 | 270 |  |  |  |  |
| *E. huxleyi* | 4 | 0.3 | 1 | 120 | 1 | 5 | 1 | 600 | 1 | 800 | 100 | 240 |
|  |  |  |  |  | 2 | 3 | 0.5 | 480 | 2 | 600 | 50 | 240 |
|  |  |  |  |  | 3 | 1 | 0.3 | 480 | 3 | 300 | 30 | 240 |
| *C. reinhardtii* | 5 | 0.5 | 0.3 | 60 | 1 | 5 | 0.3 | 210 | 1 | 800 | 50 | 180 |
|  |  |  |  |  | 2 | 3 | 0.3 | 120 | 2 | 600 | 50 | 90 |
|  |  |  |  |  | 3 | 1 | 0.1 | 120 | 3 | 400 | 50 | 60 |
| *S. cerevisiae* | 5 | 0.5 | 0.3 | 60 | 1 | 5 | 0.3 | 210 | 1 | 800 | 50 | 180 |
|  |  |  |  |  | 2 | 3 | 0.3 | 120 | 2 | 600 | 50 | 90 |
|  |  |  |  |  | 3 | 1 | 0.1 | 120 | 3 | 400 | 50 | 60 |
