# Supplementary material for: A modular platform for automated cryo-FIB workflows
Source: eLife. 2021 Dec 24;10:e70506. doi: 10.7554/eLife.70506 (PMC8769651; doi:10.7554/eLife.70506)
Supplement: Supplementary file 2. [file elife-70506-supp2.docx]

| **Grid** | **Grid**  **type** | **FIB image pixel size (nm)** | **Grid**  **position** | **Targeted lipid droplet visible in TEM? ^‡^** | **Correlated Y position post-milling relative to lamella (px)** | **Best fitting plane relative to correlated position (px)** |
| --- | --- | --- | --- | --- | --- | --- |
| 1 | Ti SiO_2_ 1/20 | 168.7 | 1 | yes | 4 | 1 |
|  |  |  | 2 | yes | 3 | 3 |
|  |  |  | 3 | yes | 11 | -2 |
|  |  |  | 4 | yes | 8 | -1 |
| 2 | Ti SiO_2_ 1/20 | 84.3 | 1 | yes | 2 | 3 |
|  |  |  | 2 | no | 7 | 2 |
|  |  |  | 3 | yes | 9 | 0 |
| 3 | Ti SiO_2_ 1/20 | 84.3 | 1 | no | 9 | 3 |
|  |  |  | 2 | no | 14 | -3 |
|  |  |  | 3 | yes | 7 | -3 |
| 4 | Au SiO_2_ 1/4 | 67.5 | 1 | N/A | -1 | N/A |
|  |  |  | 2 | no | -2 | 11 |
|  |  |  | 3 | no | -9 | 22 |
|  |  |  | 4 | no | -8 | 19 |

**^‡^** N/A = assessment could not be made due to severe condensation on lamella
